# Maternal gestational *Bifidobacterium bifidum* TMC3115 treatment shapes construction of offspring gut microbiota and development of immune system and induces immune tolerance to food allergen

**DOI:** 10.3389/fcimb.2022.1045109

**Published:** 2022-11-14

**Authors:** Ruyue Cheng, Yujie Zhang, Yang Yang, Lei Ren, Jinxing Li, Yimei Wang, Xi Shen, Fang He

**Affiliations:** ^1^ Department of Nutrition and Food Hygiene, West China School of Public Health and West China Fourth Hospital, Sichuan University, Chengdu, Sichuan, China; ^2^ Department of Research and Development, Hebei Inatural Bio-tech Co., Ltd, Shijiazhuang, Hebei, China

**Keywords:** allergy, Bifidobacterium, gut microbiota, immune function, SCFA

## Abstract

In this study we aimed to determine whether treatment with maternal *Bifidobacterium bifidum* TMC3115 could affect the composition of the gut microbiota and the development of the immune system and intestinal tract of offspring, and protect the offspring from IgE-mediated allergic disease. Pregnant BALB/c mice were gavaged with TMC3115 until delivery. Offspring were sensitized with ovalbumin from postnatal days 21 to 49. After maternal treatment with TMC3115, the microbiota of the offspring’s feces, intestinal contents, and stomach contents (a proxy for breast milk) at the newborn and weaning stages exhibited the most change, and levels of immunoglobulin in the sera and stomach contents and of splenic cytokines, as well as the mRNA levels of colonic intestinal development indicators were all significantly altered in offspring at different stages. After sensitization with ovalbumin, there were no significant changes in the levels of serum IgE or ovalbumin-specific IgE/IgG1 in the TMC3115 group; however, IgM, the expression of intestinal development indicators, and the production of fecal short chain fatty acid (SCFA) were significantly increased, as were the relative abundances of *Lactobacillus* and the *Lachnospiraceae* NK4A136 group. Our results suggested that maternal treatment with TMC3115 could have a profound modulatory effect on the composition of the gut microbiota and the development of the immune system and intestinal tissue in offspring at different stages of development, and may induce immune tolerance to allergens in ovalbumin-stimulated offspring by modulating the gut microbiota and SCFA production.

## Introduction

In recent years, the incidence of allergic diseases such as asthma, allergic rhinitis, and food allergies has been increasing in both developed and developing countries, including China (Platts-Mills, 2015). Allergic disease is a dynamic lifelong process and there is no cure at present; therefore, early prevention is particularly important. In 1989, Strachan proposed the “Hygiene hypothesis” which holds that a lack of exposure to environmental microbes in the early stages of life restricts the development and maturation of the body’s immune system, thereby increasing the risk of allergic diseases in childhood (Strachan, 1989). Evidence has since accumulated, demonstrating that the gut microbiota in early life may be a novel target for the early prevention and treatment of allergic diseases (Gensollen and Blumberg, 2017).

The period from conception to the age of two is considered to be critical for the development of the gut microbiota and the development of the immune system (Arrieta et al., 2014). Researchers have long believed that the fetus is sterile in the uterus, and that microbes first colonize the infant intestine after birth. However, current population and animal experimental studies have suggested that the first microbial exposure of newborns may occur before birth, and this exposure may have a regulatory effect on the development of the newborn’s immune system (Collado et al., 2016; Gomez de Agüero et al., 2016). Our previous study found microorganisms in the placenta and cultured live bacteria from the blood of the newborn mice and the placenta, further verifying that microbial contact with the newborn might exist before birth (Cheng et al., 2021). The maternal microbiota is considered to be the largest source of the gut microbiota of newborns, whereas the microbes of the vagina, skin, and oral cavity account for only a small proportion of the newborn gut microbiota, suggesting that the maternal microbiota has an important effect on the early gut microbiota of the offspring (Shao et al., 2019). Therefore, paying attention to the changes of the maternal microbiota during pregnancy before birth, using approaches such as probiotic intervention, is important to the development of the infant gut microbiota and immune system, and subsequent susceptibility to allergic diseases in later life.

Probiotics regulate the structure of the gut microbiota by promoting the growth of beneficial microorganisms in the intestine. Meta-analysis based on a randomized controlled double-blind trial (RCT) study suggests that the use of probiotics by mothers during pregnancy and lactation may reduce the risk of atopic dermatitis in their offspring; however, these results have a certain population heterogeneity and strain specificity (Li et al., 2019; Amalia et al., 2020), suggesting that it is necessary to explore the early preventive effect of probiotics on allergic diseases at the strain level. *Bifidobacterium*, one of the most widely studied and used probiotics, is one of the first microorganisms to colonize the human intestine, and several species, including *B. breve* and *B. bifidum*, are considered to be permanent residents. *B. bifidum* TMC3115 (TMC3115) is a probiotic strain isolated from the intestines of healthy infants (He et al., 2001). Whole genome sequencing shows that TMC3115 encodes enzymes that decompose host-derived breast milk polysaccharides (HMOs), mucins, and plant-derived polysaccharides. The complex polysaccharides that cannot be digested and absorbed by the host cells are decomposed into monosaccharides or disaccharides in the way of “Cross Feeding”, and are then provided to other bifidobacteria or intestinal bacteria, promoting symbiosis of the gut microbiota and the host (Gotoh et al., 2018). Our previous study found that supplementation with the probiotic strain TMC3115 from birth to weaning can reduce the risk of the host suffering from IgE-mediated allergic diseases in adulthood, by regulating the composition of the gut microbiota, increasing short chain fatty acid (SCFA) production, and promoting the immune system and intestinal tissue development of the host (Cheng et al., 2019). An RCT study found that TMC3115 could alleviate allergic symptoms and inflammatory responses and affect the gut microbiota composition of neonates aged 0–1 years who were allergic to cow milk proteins (Jing et al., 2020). However, the question of whether supplementation with TMC3115 during pregnancy can still exert a protective effect, and the mechanisms underlying such an effect, need further research.

In this study we explored the effects of exposure to TMC3115 during pregnancy on the maternal microbiota, and the role of the maternal microbiota in the composition of the gut microbiota of the offspring, the development of their immune system and intestinal tract, and their susceptibility to IgE-mediated allergic diseases, and we further investigated the mechanisms underlying these effects.

## Methods

### Probiotic treatment

TMC3115 was kindly provided by Hebei Inatural Biotech Co. Ltd. (Hebei, PR China). Freeze-dried living TMC3115 (2.12 × 10^11^ colony-forming unit [CFU]/g) was dissolved in sterile saline to prepare bacterial suspensions of 5 × 10^9^ CFU/mL. The daily intake of living TMC3115 was estimated as 10^9^ CFU/mouse, 0.2 mL/d, once a day.

### Animals

Fourteen pregnant specific pathogen-free BALB/c mice at gestational day 10–13 (Approval number: SCXK2015-0001) were purchased from Liaoning Changsheng Biotechnology Co., Ltd (Liaoning, PR. China), and kept in individually ventilated plastic cages at an ambient temperature of 23°C ± 1°C and humidity of 50%–70% under a 12 h light/dark cycle with free access to water and food.

**Figure 1:**
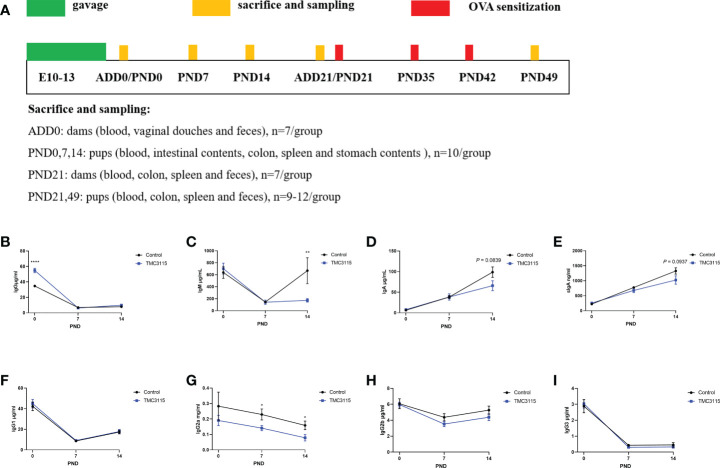
Study design and immunoglobulins of stomach contents of offspring from PND 0–14. **(A)** Experimental design. Sample sizes for microbiota analysis are clarified in the corresponding legends. **(B–I)** IgG and subclass, IgM, IgA, and sIgA levels of stomach contents of offspring from PND 0–14. *: *P <* 0.05, **: *P <* 0.01, ****: *P <* 0.0001, compared with Control. PND, postnatal day. *n* = 10/group.

The study design is shown in **Figure 1A**. Pregnant mice were randomly divided into two groups: a Control group and a TMC3115 group (*n* = 7 per group). Mice in the Control group were gavaged with saline from 1 week before and until delivery, while mice in the TMC3115 group were gavaged with a TMC3115 suspension. The organs, feces, vaginal douches, and blood of dams were collected at and after delivery day (ADD) 0 and 21, and a proportion of pups at postnatal day (PND) 0, 7, 14, and 21 were euthanized to collect blood, stomach contents (PND 0, 7, and 14), spleen, intestinal contents (PND 0, 7, and 14), colon and feces (PND 21). Then, half of the remaining pups in the two groups were intraperitoneally sensitized with ovalbumin (OVA) while the other half of the pups were not injected with any substance. All the remaining pups were sacrificed at PND49, and their organs, blood, and feces were collected.

### Sample collection

Feces of dams were collected at ADD 0 and 21 and pups at PND 21 and 49. Plastic boxes were wiped with 75% ethanol, the mice were placed into these boxes, and more than 200 mg of naturally excreted feces were collected from each mouse within several hours.

Vaginal douches and blood of dams at ADD 0 were collected within 12 hours after the dams gave birth. Dams were placed into a 50 mL centrifuge tube, and their tails were exposed to the outside of the centrifuge tube and secured with tape. Then, the skin near the vagina and the tails of dams were wiped twice with 75% ethanol, with the dams ventral side up. A pipette was used to aspirate 50 μL of sterile saline, and the tip was carefully inserted into one side of the dams’ vagina, and then pipetting was repeated several times, avoiding saline leakage and contamination, and then the rinsing fluid was sucked out and placed in a 1.5 mL centrifuge tube. Vaginal douches were collected from the other side of the dams’ vaginas using the same method. A sterile surgical blade was used to make a small incision 2–3cm from the base of the tail, and 100 μL of blood was collected in a 1.5 mL centrifuge tube. Finally, a cotton ball was pressed on the wound for two minutes to stop the bleeding and keep the dams alive.

The blood of dams at ADD 21 and of pups at PND 21 and PND 49 were collected by removing the eyeballs after the mice were euthanized by inhalation of carbon dioxide. The blood of pups at PND 0, 7, and 14 was collected by beheading after they were euthanized by inhalation of carbon dioxide. The colon and spleen of mice were collected under sterile conditions after the mice were sacrificed.

The stomach and intestinal contents of pups were collected under sterile conditions at PND 0, 7, and 14, after the pups were sacrificed. The stomach contents were considered to be a proxy for breast milk (Nyangahu et al., 2018), because it was difficult to collect milk from the nipples of the dams, while avoiding contamination. The intestinal contents was considered to be a proxy for feces, because the pups were too young for the collection of. Briefly, the whole stomach was collected using sterile scissors and tissue forceps, and placed into a sterile 1.5 mL centrifuge tube. Then, the stomach tissue was bluntly dissected using tissue forceps, and the stomach contents of the pups were left in the tubes. The whole intestine of pups were collected under sterile conditions and placed in a sterile plastic plate. The intestinal contents were expelled from the intestinal lumen using a sterile curved forceps along the entire intestinal tract. Two hundred microliters of sterile PBS was added to the plate, and the viscous intestinal contents were collected by repeatedly pipetting and rinsing with a pipette. The resulting mixture was placed into a 1.5 mL centrifuge tube. The collected intestinal contents mixture actually contained the contents in the small intestine and colon, thus the microbiota found in the intestinal contents was named as small intestinal and colonic microbiota in the following text.

### OVA sensitization

The OVA sensitization procedure was performed as previously described, with some modifications (Cheng et al., 2019). Briefly, pups were injected intraperitoneally with 40 μg of OVA and 4 mg of Imject Alum (Sigma-Aldrich, St. Louis, MO, USA) suspension on PND 21, 35, and 42.

### Total RNA extraction from animal tissue and quantitative reverse transcription-polymerase chain reaction

Total RNA was extracted from spleen and colon using a commercial kit (Foregene, Chengdu, PR. China) in accordance with the manufacturer’s instructions. Quantitative reverse transcription-polymerase chain reaction (RT-PCR) was performed as previously described, with minor modifications (Cheng et al., 2020). Briefly, complementary DNA (cDNA) was synthesized using iScript™ cDNA synthesis kits (Bio-Rad, Hercules, CA, USA), and the quantification of cDNA was performed using SsoAdvanced™ Universal SYBR^®^ Green Supermix (Bio-Rad). The quantitative PCR reaction system consisted of 5 μL of 1x SsoAdvanced™ Supermix, 0.5 μL of 100 μM forward primer, 0.5 μL of 100 μM reverse primer, 1 μL of cDNA (100 ng to 100 fg), and 3 μL of double-distilled water (ddH2O). The cycling conditions were as follows: initial denaturation at 98°C for 30 s, followed by 40 cycles of denaturation at 98°C for 15 s, and annealing/extension at 60°C for 30 s. β-actin was used as the reference gene, and 2^−△△ct^ calculation was used to determine the relative levels of the mRNA of the targeted gene. Primer sequences are listed in **Table S1**. All primers were synthesized by Shanghai Sangon Biotech Co., Ltd. (Shanghai, China PR).

### Hematoxylin-eosin staining

Colons were collected from pups at PND 21. One centimeter of each sample was cut and fixed with 10% neutral phosphate-buffered saline formalin for 24 h, and then stained with hematoxylin-eosin (HE). Optical microscopic images were inspected by a pathologist blinded to the experimental design. As previously described (Cheng et al., 2017), an optical microscope (Olympus Corporation, Tokyo, Japan) was used to image the intestinal tissues. The fields of view were randomly selected, and included non-necrosis, hemorrhage, and non-specific staining marginal areas. A charged coupled device was used to collect high-quality images. Ten areas were measured from each of the upper, lower, left, right, and middle of each slice, and the parameters of 50 colonic crypts in total on each slice were measured.

### Enzyme-linked immunosorbent assay

The levels of IgG, IgM, IgA, sIgA, IgG1, IgG2a, IgG2b, and IgG3 in serum and the supernatant of stomach contents, and sIgA from the supernatants of feces and intestinal contents were detected using enzyme-linked immunosorbent assay (ELISA) with commercial kits (Elabscience, Wuhan, PR. China). Aliquots of 10 mg of stomach contents were added to 90 μL of sterile PBS and centrifuged for 3 min at 6000 g to obtain supernatants. Feces (50 mg) were mixed with 200 μL of sterile PBS, vortexed, and then centrifuged for 3 min at 6000 g to obtain supernatants. Serum IgE was determined *via* ELISA using mouse IgE ELISA kits (Invitrogen, Thermo Fisher Scientific, Inc., MA, USA). Serum OVA-specific IgE and IgG1 were detected using Anti-Ovalbumin IgE (mouse) ELISA kits and IgG1 (mouse) ELISA kits (Cayman Chemical, MI, USA). The serum cytokines IL-1β, IL-4, IL-5, IL-6, IL-10, IL-12p70, IL-13, IL-17A, and IFN-γ were measured using Luminex (R&D Systems Inc., MN, USA). Assays were performed according to the manufacturer’s instructions and read using a Multiskan™ GO microplate spectrophotometer (Thermo Fisher Scientific, Inc., MA, USA) and a Luminex 200™ multiplexing instrument (Merck Millipore, MA, USA).

### DNA extraction from intestinal contents, feces, vaginal douches and stomach contents

Total DNA was extracted from mouse intestinal contents and feces (100 mg) of offspring at PND 0, 7, 14, 21, and 49, maternal feces at ADD 0 and 21, stomach contents at PND 0, 7, and 14, and maternal vaginal douches at ADD 0, using the TIANamp Stool DNA Kit (Tiangen Biotech Co. Ltd., Beijing, PR China) in accordance with the manufacturer’s instructions.

### Amplification and sequencing of 16S rRNA encoding gene sequences

The 5’ ends of the primers were tagged with barcodes specific to each sample and sequencing universal primers. PCR amplification was performed in a total volume of 25 μL of reaction mixture containing 50 ng of DNA, 12.5 μL of Phusion Hot start flex 2X Master Mix (NEB, M0536L), 2.5 μL of each of forward primer 338F 5’-ACTCCTACGGGAGGCAGCAG-3’ and reverse primer 806R 5’-GGACTACHVGGGTWTCTAAT-3’ (16S V3-V4 primer), and ddH_2_O was used to adjust the volume. The PCR conditions were 98°C for 30 s, followed by 35 cycles of 98°C for 10 s, 54°C for 30 s, and 72°C for 45 s, and then 72°C for 10 min. The PCR products were separated using 2% agarose gel electrophoresis, purified with AMPure XT beads (Beckman Coulter Genomics, Danvers, MA, USA), and then quantified using a Qubit (Invitrogen, USA). Amplicon pools were prepared for sequencing, and the size and quantity of the amplicon library were assessed using an Agilent 2100 Bioanalyzer (Agilent Technologies, Santa Clara, CA, USA) and the Library Quantification Kit for Illumina (Kapa Biosciences, Woburn, MA, USA), respectively. A PhiX Control library (v3) (Illumina) was combined with the amplicon library (expected at 30%). The libraries were sequenced on an Illumina MiSeq Instrument (Illumina Inc., San Diego, CA, USA) using the 300-base pair (bp) paired-end protocol with the standard Illumina sequencing primers.

### Bioinformatics

Briefly, raw FASTQ files were generated using Illumina’s bcl2fastq (v1.8.4) software. Trimmomatic (v0.36) was used to remove the barcode and primer sequences, and paired-end reads were then merged using FLASH (v1.2.11). The two-terminal valid and high-quality sequences were then spliced using the QIIME2-DADA2 process, which required sequence overlap of at least 10 bp and allowed a maximum of two base mismatches. The Amplicon Sequence Variants (ASVs) were constructed and taxonomic assignment was performed based on the SILVA Living Tree Project v138 (LTPv138) database (16S V3-4). Multiple sequences were aligned and a phylogenetic tree was constructed. The phylogenetic tree, ASVs abundance table, alpha diversity (ACE, Chao1, observed features, Shannon, Simpson), and beta diversity (Bray–Curtis, weighted and unweighted UniFrac) were calculated using QIIME2. The pairwise difference between the mean values species richness and the diversity index was measured using the Wilcox test (R program). Microbiota community composition was shown at phylum and genus level. All plots were visualized within the R program (v3.4.1).

### Detection of fecal SCFA

Fecal SCFA (acetic, propionic, and butyric acid) were detected using gas chromatography. Feces (100 mg) were mixed with 400 μL of ether, 100 μL of internal standard (4-methyl valeric acid), and 100 μL of 15% phosphoric acid, vortexed, and then centrifuged for 10 min at 4°C at 13400 g. Sample supernatant (1 μL) was analyzed using an Agilent 7890A (Agilent Technologies, Inc.).

### Statistical analysis

Statistical analysis was performed using GraphPad Prism 8.0.1 (GraphPad Software, San Diego, California USA, www.graphpad.com). Data are expressed as the mean ± standard error of the mean. *Student’s t*-tests or Mann–Whitney U tests were used for pairwise comparisons. A probability (*P*) value < 0.05 was considered significant. All statistical tests were two-tailed.

## Results

### Effect of TMC3115 on maternal immune function, vaginal and fecal microbiota, and SCFA production

After delivery day 0, there was a significant decrease in the levels of serum IgG1, IgG3, and fecal sIgA in dams treated with TMC3115 in comparison with those in the Control group, while the levels of serum IgM tended to decrease (**Figure S1**). After delivery day 21, the levels of serum immunoglobulin and fecal sIgA of dams treated with TMC3115 were similar to those of the Control group (**Figure S2A**). However, compared with those in the Control group, the mRNA expression of splenic IL-8, IL-10, and IL-17A in dams treated with TMC3115 was significantly increased; moreover, the expression of TNF-α and TGF-β1 showed a tendency to be increased (**Figures S2B–H**).

After delivery day 0, the alpha diversity of dams treated with TMC3115 did not significantly change in comparison with that of the Control (**Figure S3A**). However, the fecal microbiota community was altered to some extent, and Principal Coordinates Analysis (PCoA) analysis showed two clear clusters (**Figures S3B–D**). At the phylum and genus level, the relative abundances of *Proteobacteria*, *Deferribacteres, Helicobacter*, *Mucispirillum*, and *Prevotella* in the TMC3115 group were significantly lower than those in the Control group, while the relative abundance of *Rikenella* in the TMC3115 group was significantly higher (**Table S2**). The level of fecal acetic acid from dams treated with TMC3115 was significantly decreased compared with that in the Control group (**Figure S3E**).

After delivery day 21, the Simpson index of the TMC3115-treated group was significantly lower than that of the Control group, while the Shannon index of the TMC3115-treated group tended to decrease (**Figure S3F**). The fecal microbiota composition was altered by TMC3115 treatment during pregnancy, as demonstrated by significantly decreased abundance of *Alistipes* and increased abundances of *Lactobacillus*, *Roseburia*, and *Staphylococcus*; PCoA also showed two clusters separated by the axis, with several overlapping areas (**Figures S3G–I** and **Table S2**). The fecal acetic and propionic acid levels in the TMC3115-treated group were significantly lower than those in the Control group (**Figure S3J**).

On the day after dams gave birth, vaginal douches were collected and sequenced to observe changes to the vaginal microbiota. The ACE, Chao1, and observed OTUs of the TMC3115 group were significantly lower than those of the Control group, while the Shannon and Simpson indexes also tended to decrease (**Figure S4A**). The Bray–Curtis distance was used to compare the beta diversity between groups, and two clusters were separated using PCoA analysis (**Figure S4B**). At the phylum level, the relative abundances of *Firmicutes* and *Desulfobacterota* in the TMC3115 group were significantly decreased, while the abundance of *Proteobacteria* was significantly increased when compared with those of the Control group (**Figure S4C** and **Table S2**). At the genus level, the relative abundances of *Oscillibacter* A2, *Lachnospiraceae* UCG.006, *Clostridia vadin* BB60 group, *Desulfovibrio*, *Lachnospiraceae* NK4A136 group, *Citrobacter* undefined*, Lachnospiraceae*, and *Lactobacillus* in the TMC3115 group were significantly decreased when compared with those in the Control group; however, the abundances of *Escherichia, Shigella*, *Bifidobacterium*, and *Psychrobacter* were significantly increased (**Figure S4D** and **Table S2**).

### Alterations to immunoglobulin and the microbiota of the stomach contents

Between PND 0 and PND 14, the level of IgG and associated subclasses, and the IgM level in stomach contents gradually decreased, while the levels of IgA and sIgA gradually increased (**Figures 1B–I**). At PND 0, the IgG content of the stomach contents in the TMC3115 group was significantly higher than that in the Control group (**Figure 1B**). The IgG2a content at PND 7 and PND 14, and the IgM content at PND 14 in the TMC3115 group were significantly lower than those in the Control group (**Figures 1C, G**). The IgA and sIgA contents at PND 14 in the TMC3115 group tended to be lower than those in the Control group (**Figures 1D, E**).

**Figure 2:**
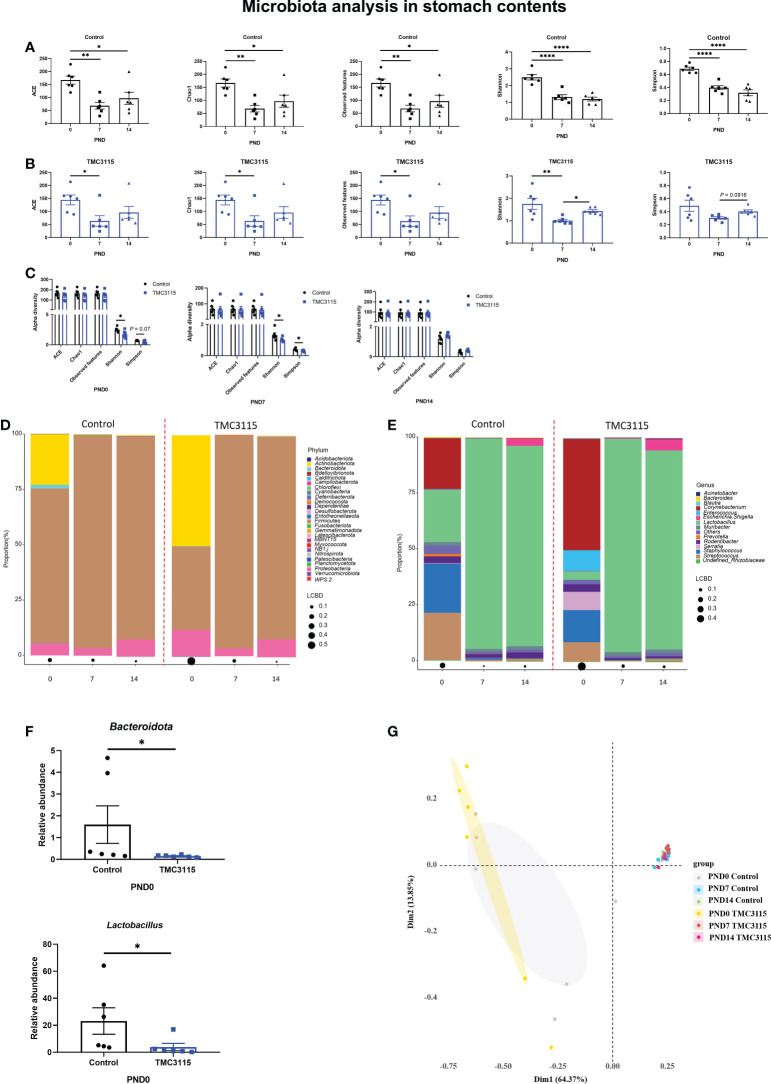
Diversity and composition of stomach content microbiota of offspring from PND 0–14. **(A, B)** Changes of alpha diversity of the microbiota of the stomach contents of offspring from PND 0–14 in two groups. **(C)** Differences of alpha diversity between groups. **(D, E)** Microbial community of the stomach contents of offspring at phylum and genus levels. **(F)** Significantly altered bacteria between groups. (h) PCoA analysis based on Bray-Curtis distance. *: *P <* 0.05, **: *P <* 0.01, ****: *P <* 0.0001, compared with Control. PND, postnatal day. *n* = 6/group.

In the Control group, the alpha diversity at PND 0 was significantly higher than those at PND 7 and PND 14 (**Figure 2A**). Similarly, in the TMC3115 group, the alpha diversity, including ACE, Chao1, observed OTUs, and Shannon index at PND 0 were significantly higher than those at PND 7; the Shannon index at PND 14 was also significantly higher than that at PND 7 (**Figure 2B**). At PND 0 and PND 7, the Shannon and Simpson indexes of the TMC3115 group were significantly lower than those of the Control group; however, there were no significant differences in alpha diversity at PND 14 between the groups (**Figure 2C**).

At PND 0, *Firmicute*s, *Actinobacteriota*, and *Proteobacteria* were the three predominate phyla, and *Lactobacillus*, *Staphylococcus*, *Streptococcus*, and *Corynebacteriu*m were the four predominate genera in the stomach contents. However, the relative abundances of *Actinobacteriota*, *Staphylococcus*, *Streptococcus*, and *Corynebacteriu*m gradually decreased, while the relative abundances of *Firmicute*s and *Lactobacillus* largely increased. Therefore, *Firmicute*s and *Proteobacteria* became the predominate phyla and *Lactobacillus* became the only predominate genus in stomach contents at PND 7 and PND 14 (**Figures 2D, E**). Only the abundances of *Bacteroidota* and *Lactobacillus* in the TMC3115 group at PND 0 were significantly lower than those in the Control group (**Figure 2F**). No significant differences in abundance were found between the groups at PND 7 and PND 14. Consistently, PCoA analysis indicated that the microbiota community was similar at PND 7 and PND 14 between the Control and TMC3115 groups, which were gathered into one cluster but clearly separated from samples at PND 0. The samples in the Control and TMC3115 groups were gathered into two clusters with several overlapping areas (**Figure 2G**).

### Alterations to immune function, intestinal epithelial cells, and barrier function development of offspring

**Figure 3:**
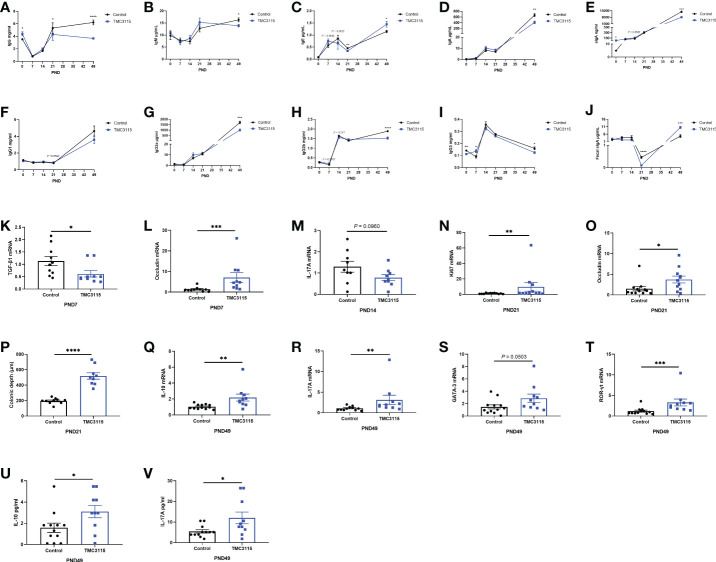
Levels of serum immunoglobulins and cytokines, mRNA expression of splenic cytokines, and intestinal tissue development of offspring from PND 0–49. **(A–J)** Significant changes in serum immunoglobulins and fecal sIgA levels of offspring from PND 0–49 between two groups. **(K–L)** Significant changes in splenic cytokines and colonic occludin mRNA expression of offspring at PND 7 between two groups. **(M, O)** Significant changes in splenic cytokines mRNA expression of offspring at PND 14 between two groups. **(N–P)** Significant changes in colonic Ki67, occludin mRNA expression, and colonic depth of offspring at PND 21 between two groups. **(Q–V)** Significant changes in splenic cytokines, mRNA expression of transcriptional factors, and serum cytokines of offspring at PND 49 between two groups. *: *P <* 0.05, **: *P <* 0.01, ***: *P <* 0.001, ****: *P <* 0.0001, compared with Control. PND, postnatal day. *n* = 9–12/group.

The levels of serum immunoglobulin and fecal sIgA dynamically changed with age. At PND 0, the serum IgG and sIgA levels in the TMC3115-treated group were significantly higher than those in the Control group, while the IgG3 levels were lower than those in the Control group (**Figures 3A, E, I**).

At PND 7, the levels of serum IgG3 in the TMC3115 group were significantly higher than those in the Control group while those of IgE and IgG2b only tended to be higher (**Figures 3C, H, I**). Compared with that in the Control group, the level of mRNA of splenic TGF-β1 was significantly decreased, and the mRNA level of colonic occludin was significantly increased (**Figures 3K, L**).

At PND 14, the levels of serum IgE and IgG2b in the TMC3115 group tended to be lower, while that of sIgA tended to be higher than those in the Control group (**Figures 3C, E, H**). The mRNA expression of splenic IL-17A tended to decrease when compared with that of the Control group (**Figure 3M**).

At PND 21, the levels of serum IgG and IgE and fecal sIgA in the TMC3115 group were significantly lower than those in the Control group, while the levels of serum IgG1 levels tended to be higher (**Figures 3A, C, F, J**). Colonic Ki67 and occludin mRNA expression and the colonic depth were significantly increased compared with those in the Control group (**Figures 3N–P**).

At PND 49, the levels of serum IgG, IgM, IgA, sIgA, IgG2a, IgG2b, and IgG3 in the TMC3115 group were significantly lower, and levels of serum IgE and fecal sIgA were significantly higher than those in the Control group (**Figures 3A–J**). The splenic mRNA expression of IL-10, IL-17A, and ROR-γt, and levels of serum IL-10 and IL-17A in the TMC3115 group were significantly increased compared with those in the Control group, while the splenic GATA-3 mRNA expression in the TMC3115 group tended to be increased (**Figures 3Q–V**).

### Alterations of fecal, small intestinal, and colonic microbial composition and SCFA production of offspring

**Figure 4:**
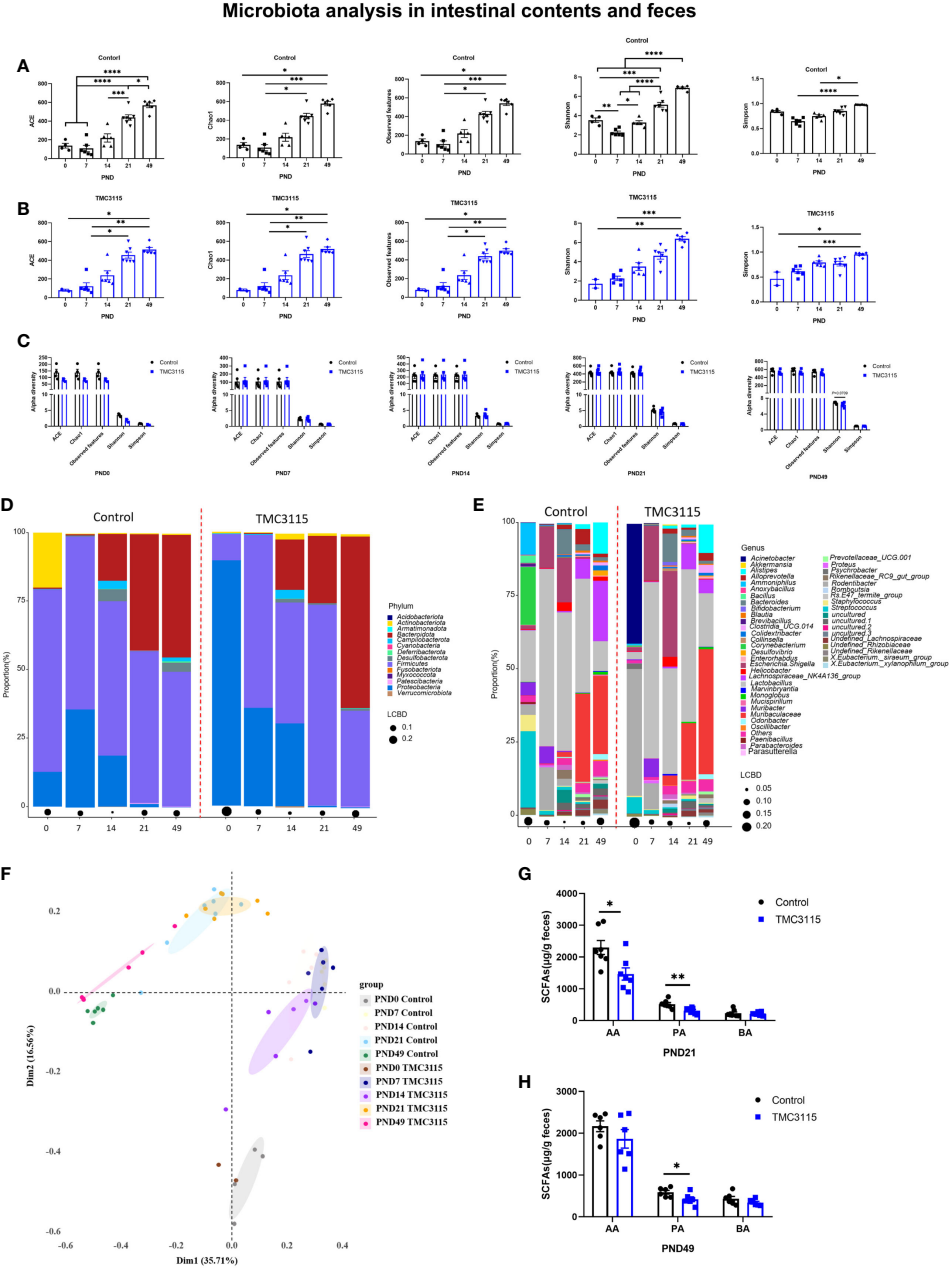
Diversity and composition of the fecal, small intestinal and colonic microbiota of offspring from PND 0–49. **(A, B)** Changes of alpha diversity of fecal, small intestinal and colonic microbiota of offspring from PND 0–49 in two groups. **(C)** Differences of alpha diversity of the fecal, small intestinal and colonic microbiota of offspring between groups. **(D, E)** Fecal, small intestinal and colonic microbial community of offspring at phylum and genus levels. **(F)** PCoA analysis based on Bray-Curtis distance. **(G, H)** Fecal SCFA production of offspring at PND 21 and PND 49. *: *P <* 0.05, **: *P <* 0.01, ***: *P <* 0.001, ****: *P <* 0.0001, compared with Control. PND: postnatal day. AA, acetic acid; PA, propionic acid; BA, butyric acid. PND 0, Control group, *n* = 4, TMC3115 group, *n* = 2; PND 7 and PND 14, *n* = 6/group; PND 21, *n* = 7/group; PND 49, *n* = 6/group.

In the Control group, the alpha diversity (including ACE, Chao1, Observed OTUs, Shannon, and Simpson index) gradually but significantly increased with age. However, at PND 7, the alpha diversity was the lowest among the time points, and the Shannon index was significantly lower than at other time points (**Figure 4A**). Similarly, in the TMC3115 group, the alpha diversity gradually but significantly increased with age (**Figure 4B**). There were no significant differences found in alpha diversity between groups at any of the time points, although the Shannon index in the TMC3115 group tended to be lower than that in the Control group (**Figure 4C**).

In the Control group at PND 0, the phyla predominating in the small intestinal and colonic microbiota were *Proteobacteria*, *Firmicutes*, and *Actinobacteriota*, while the predominate genera were *Lactobacillus*, *Corynebacterium*, *Streptococcus*, and *Ammoniphilus*. In the TMC3115-treated group, the two phyla predominating the small intestinal and colonic microbiota were *Proteobacteria* and *Firmicutes*, while *Acinetobacter* and *Rodentibacter* were the two predominate genera (**Figures 4D, E**). With age, the relative abundances of *Proteobacteria* and *Actinobacteriota* gradually decreased, while the abundance of *Bacteroidota* gradually increased in the two groups. The relative abundances of the predominate genera at PND 0 gradually decreased, and several newly colonized genera predominated with increasing age. At PND 7, the two predominate phyla in both groups were *Proteobacteria* and *Firmicutes*, while the three predominate genera were *Lactobacillus*, *Escherichia-Shigella*, and *Rodentibacter*. At PND 14, the phyla *Bacteroidota*, *Proteobacteria*, and *Firmicutes* and genera *Lactobacillus*, *Escherichia-Shigella*, and *Bacteroides* predominated in both groups. At PND 21 and 49, the phyla *Bacteroidota* and *Firmicutes* and genera *Lactobacillus*, *Muribaculaceae*, and *Lachnospiraceae* NK4A136 group were predominate in both groups.

At PND 0, the relative abundances of *Proteobacteria*, *Acinetobacter*, and *Rodentibacter* in the TMC3115 group were significantly increased compared with those in the Control group, whereas those of *Firmicutes*, *Bacteroidota*, *Lactobacillus*, *Corynebacterium*, and *Staphylococcus* were significantly decreased, and the abundances of *Actinobacteriota* and *Streptococcus* were slightly decreased. At PND 21, the relative abundances of *Patescibacteria* and *Prevotellaceae* UCG.001 were significantly decreased, and those of *Bacteroidota*, *Alistipes*, and *Muribaculaceae* tended to be decreased, whereas the relative abundances of *Firmicutes*, *Desulfobacterota*, *Lactobacillus*, and *Enterorhabdus* tended to be increased. At PND 49, the relative abundances of *Actinobacteriota*, *Bacteroidota*, *Rikenellaceae* RC9 gut group, *Clostridia* UCG 014, and *Muribaculaceae* were significantly increased, whereas those of *Desulfobacterota*, *Firmicutes*, undefined *Lachnospiraceae*, *Lachnospiraceae* NK4A136 group, *Eubacterium siraeum* group, and *Colidextribacter* were significantly decreased (**Table 1**).

**Table 1:** Mean relative abundance of microbiota in feces and intestinal contents of offspring from PND 0-49 at phylum and genus level (%).

Stage	Phylum/Genus	Control	TMC3115
PND0	*Proteobacteria*	13.44	89.74^***^
	*Firmicutes*	66.65	9.43^**^
	*Bacteroidota*	0.46	0.04^*^
	*Actinobacteriota*	19.14	0.55 (*P* = 0.071)
PND0	*Lactobacillus*	18.11	2.46^*^
	*Staphylococcus*	5.43	0.49^*^
	*Corynebacterium*	18.96	0.38 (*P* = 0.072)
	*Streptococcus*	25.22	5.38 (*P* = 0.072)
PND21	*Patescibacteria*	0.33	0.10^*^
	*Firmicutes*	54.93	72.95 (*P* = 0.054)
	*Bacteroidota*	42.87	24.84 (*P* = 0.056)
	*Desulfobacterota*	0.23	0.53 (*P* = 0.078)
PND21	*Prevotellaceae.UCG.001*	1.16	0.15^*^
	*Lactobacillus*	38.34	51.80 (*P* = 0.071)
	*Alistipes*	1.77	1.07 (*P* = 0.084)
	*Enterorhabdus*	0.23	0.48 (*P* = 0.092)
	*Muribaculaceae*	30.17	19.60 (*P* = 0.093)
PND49	*Actinobacteriota*	0.43	0.84^**^
	*Bacteroidota*	45.10	62.65^*^
	*Desulfobacterota*	3.25	0.70^*^
	*Firmicutes*	49.02	34.99^*^
PND49	*Undefined.Lachnospiraceae*	3.06	1.07^**^
	*Lachnospiraceae.NK4A136.group*	20.42	4.72^**^
	*Rikenellaceae.RC9.gut.group*	0.28	1.28^**^
	*Eubacterium.siraeum.group*	1.30	0.34^**^
	*Clostridia.UCG.014*	1.07	2.66^*^
	*Muribaculaceae*	27.20	42.77^*^
	*Colidextribacter*	1.65	0.94^*^

PND, postnatal day; *: compared with Control, P < 0.05; **: compared with Control, P < 0.01. ***: compared with Control, P < 0.001. Welch’s T test was used for pairwise comparison. PND0, Control group, n = 4, TMC3115 group, n = 2; PND21, n = 7/group; PND49, n = 6/group.

There were four clusters separated *via* PCoA analysis based on Bray–Curtis distance. These groups included samples taken at PND 0, 7, 14, 21, and 49. Samples in the two groups at PND 0 and PND 49 were clearly separated, while samples in the two groups at PND 7, 14, and 21 overlapped (**Figure 4F**). The levels of fecal acetic and propionic acid at PND 21 and of propionic acid at PND 49 in the TMC3115 group were significantly lower than those in the Control group (**Figures 4G, H**).

### Susceptibility and immune response to IgE-mediated allergic disease and intestinal development of offspring

**Figure 5:**
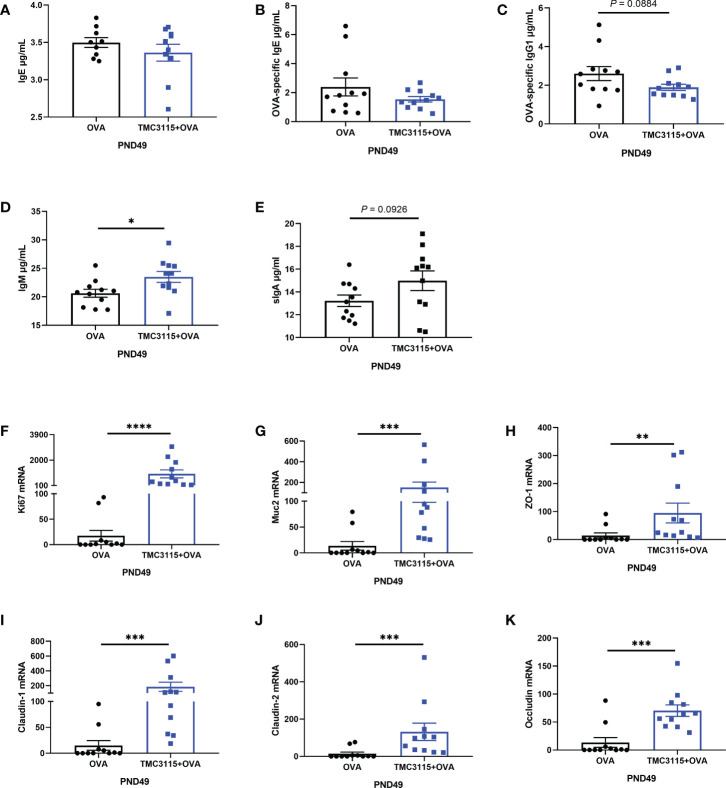
Serum immunoglobulins, mRNA expression of splenic cytokines, and intestinal tissue development of offspring after OVA stimulation. **(A–E)** Serum IgE, OVA-specific IgE/IgG1, IgM, and sIgA levels of offspring after OVA stimulation at PND 49. **(F-K)** Colonic Ki67, Muc2, and mRNA expression of tight junction proteins. *: *P <* 0.05, **: *P <* 0.01, ***: *P <* 0.001, ****: *P <* 0.0001, compared with Control. PND, postnatal day. *n* = 11/group.

After sensitization by OVA, there were no significant changes in the levels of serum IgE and OVA-specific IgE of offspring at PND 49 in the TMC3115 group compared with the Control group (**Figures 5A, B**). However, the levels of serum OVA-specific IgG1 in the TMC3115 group tended to be decreased in comparison with those in the Control group (**Figure 5C**). Moreover, the level of serum IgM in the TMC3115 group was significantly higher than that in the Control group, while that of serum sIgA also tended to be higher (**Figures 5D, E**). The colonic mRNA expression of Ki67, Muc2, ZO-1, claudin-1, claudin-2, and occludin in the TMC3115 group was significantly higher than that in the Control group (**Figures 5F–K**).

### Response of fecal microbiota and alterations of SCFA to OVA stimulation of offspring

**Figure 6:**
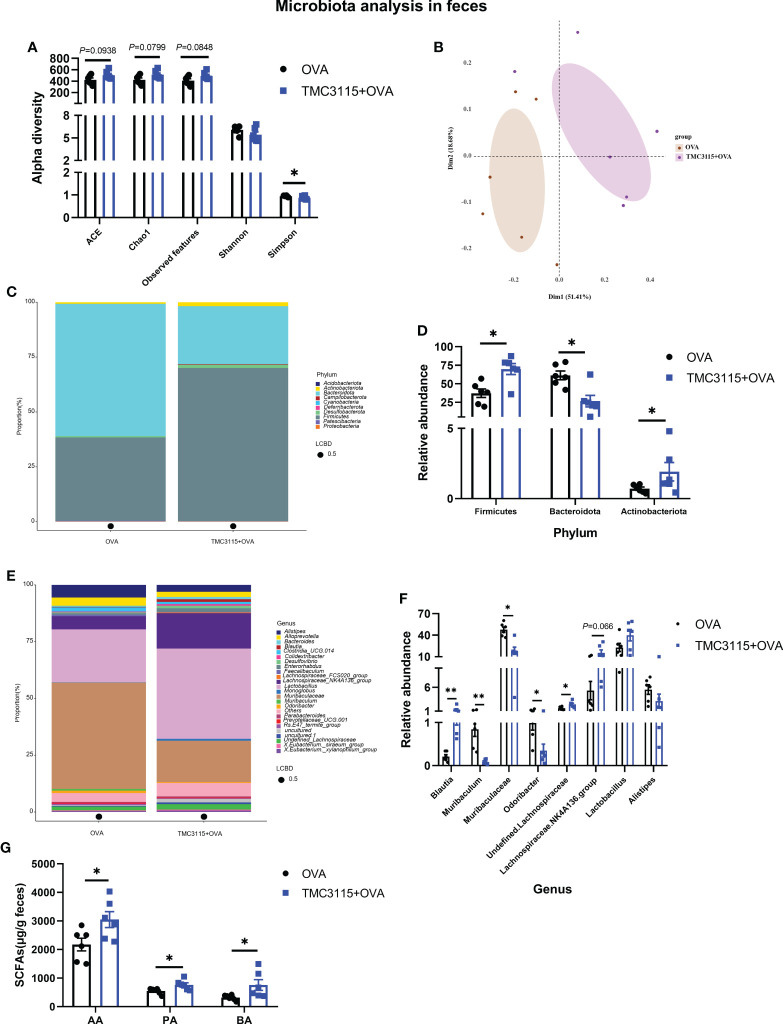
Diversity and composition of fecal microbiota of offspring after OVA stimulation at PND 49. **(A)** Alterations of fecal microbial alpha diversity of offspring between groups. **(B)** PCoA analysis based on Bray-Curtis distance. **(C, D)** Fecal microbial community of offspring at the phylum level and significantly changed phyla between groups. **(E, F)** Fecal microbial community of offspring at the genus level and significantly changed genera between groups. **(G)** Fecal SCFA production of offspring after OVA stimulation at PND 49. *: *P <* 0.05, compared with Control. **: compared with Control, P <0.01. PND, postnatal day; AA, acetic acid; PA, propionic acid; BA, butyric acid. *n* = 6/group.

After sensitization by OVA, the ACE, Chao1 index, and observed OTUs of the TMC3115 group tended to be higher than those of the Control group, while the Simpson index of the TMC3115 group was significantly lower (**Figure 6A**). Samples in the two groups were clearly separated into two clusters *via* PCoA (**Figure 6B**). The phyla *Bacteroidota*, *Firmicutes*, and genera *Lactobacillus*, *Muribaculaceae*, and *Lachnospiraceae* NK4A136 group predominated in both groups (**Figures 6C, E**). Compared with the Control group, the relative abundances of the phyla *Actinobacteriota* and *Firmicutes*, and the genera *Blautia* and unidentified *Lachnospiriaceae* in the TMC3115 group were significantly increased, while the abundance of the *Lachnospiraceae* NK4A136 group tended to be increased. However, the relative abundances of the phylum *Bacteroidota* and the genera *Muribaculum*, *Muribaculuceae*, and *Odoribacter* in the TMC3115 group were significantly decreased (**Figures 6D, F**). The fecal acetic, propionic, and butyric acid levels in the TMC3115 group were significantly higher than those in the Control group (**Figure 6G**). All the main findings of this manuscript have been summarized in **Table 2**.

**Table 2:** Summary of main findings.

Time points		Main findings in TMC3115 group
Dams	ADD0	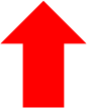 : vaginal *Proteobacteria*, *Bifidobacterium*
		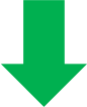 : serum IgG1, IgG3 level; fecal sIgA level; fecal *Proteobacteria*; fecal AA level; vaginal alpha diversity; vaginal *Firmicutes* and *Lactobacillus*
	ADD21	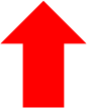 : splenic IL-8, IL-10, and IL-17A mRNA expression; fecal *Lactobacillus*
		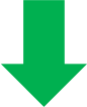 : simpson index of fecal microbiota; fecal AA and PA level;
Pups	PND0	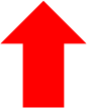 : serum IgG, sIgA level IgG level in stomach contents; *Proteobacteria* was predominate in stomach contents; small intestinal and colonic *Proteobacteria* and *Acinetobacter*
		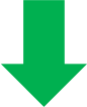 : serum IgG3 level; small intestinal and colonic *Firmicutes*, *Bacteroidota*, *Lactobacillus;* alpha diversity and *Bacteroidota* and *Lactobacillus* in stomach contents
	PND7	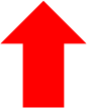 : serum IgG3 level; colonic occludin mRNA expression
		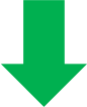 : stomach contents IgG2a level; splenic TGF-β1 mRNA expression; alpha diversity of microbiota in stomach contents
		No significance: microbiota diversity and composition in stomach contents; small intestinal and colonic microbiota composition of intestinal contents
	PND14	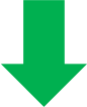 : stomach contents IgG2a and IgM level
		No significance: microbiota diversity and composition in intestinal contents and stomach contents
	PND21	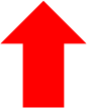 : colonic Ki67 and occludin mRNA expression; colonic depth; fecal *Firmicutes* and *Lactobacillus*
		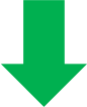 : serum IgG, IgE level; fecal sIgA level; fecal *Prevotellaceae* UCG.00; fecal AA and PA level
	PND49	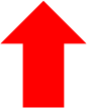 : serum IgE, IL-10, IL-17A level; splenic IL-10, IL-17A, and ROR-γt mRNA expression; fecal sIgA level; fecal *Bacteroidota* and *Muribaculaceae*
		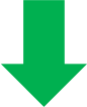 : serum IgG, IgM, IgA, sIgA, IgG2a, IgG2b, and IgG3 level; fecal *Firmicutes* and *Lachnospiraceae* NK4A136 group; fecal PA level
	PND49 (OVA-stimulated)	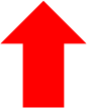 : serum IgM level; colonic Ki67, Muc2, ZO-1, claudin-1, claudin-2, and occludin mRNA expression; fecal *Firmicutes, Lactobacillus* and *Lachnospiraceae* NK4A136 group; fecal AA, BA and PA level
		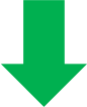 : simpson index, fecal *Bacteroidota*
		No significance: serum IgE and OVA-specific IgE/IgG1

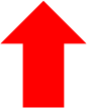
 increased indicators in TMC3115 group when compared with the Control group; 
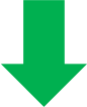
 decreased indicators in TMC3115 group when compared with the Control group. Negative results were not totally shown in this table.

## Discussion

Pregnancy has an important effect on the gut microbiota composition. During this period, the structure of the gut microbiota is quite different from that found in non-pregnant individuals. There is a decline in diversity and the amount of butyric acid producing bacteria, and an increase in the abundance of *Proteobacteria* and *Actinobacteria*, as adaptations to the physiological pro-inflammatory response occurring during pregnancy (Koren et al., 2012). In the present study, gestational TMC3115 treatment did not alter the diversity of maternal gut microbiota, and the microbial composition remained relatively stable. However, gestational TMC3115 treatment reduced the levels of maternal SCFA, even when the treatment was discontinued for 21 days. SCFA have been shown to modify immune systems. A significant increase in the levels of mRNA for splenic cytokines after gestational TMC3115 treatment indicated that the immune function of mothers was altered to some extent. In this study, alteration of the maternal gut microbiota by gestational TMC3115 treatment was not apparent, while there may be regulation of the mother’s immune function through reduced SCFA production.

We observed changes in the vaginal microbiota after gestational TMC3115 administration. The vaginal microbiota has been shown to be dominated by *Lactobacillus*, which could affect the composition of the neonatal gut microbiota (Greenbaum et al., 2019). The vaginal microbiota was markedly changed, with significantly decreased alpha diversity and abundance of *Firmicutes* and *Lactobacillus*, and significantly increased abundance of *Proteobacteria*; these changes in microbial composition were also observed in the gut microbiota of neonates. Gestational administration of TMC3115 significantly increased the relative abundance of vaginal *Bifidobacterium*, which was not found in the gut microbiota of neonates, indicating the TMC3115 might promote the vertical transmission of bifidobacteria from mother’s intestine to vagina as the evolutionary trait of bifidobacteria and the low diversity of vaginal microbiota and the low pH value within vagina (Wang et al., 2020; Stuivenberg et al., 2022). These results indicated that gestational TMC3115 treatment could significantly alter the microbiota of the mothers’ vaginas and the pups’ intestines.

The maternal gut microbiota is also important in the composition of the breast milk microbiota and the gut microbiota of the offspring (Perez et al., 2007; de Andrés et al., 2017). In this study, the gut and stomach contents microbiota of pups at PND 0 were the most altered by gestational TMC3115 treatment, and were comparable with those at PND 7 and PND 14. At PND 0, the abundance of *Proteobacteria* was significantly increased and that of *Lactobacillus* and *Firmicutes* significantly decreased, in the vagina, stomach contents, and small intestinal and colonic contents, whereas the converse was found in the maternal gut microbiota at the same time point. These results were consistent with our previous birth cohort study (Shen et al., 2019); indicating that the “window of opportunity” period for the construction of the neonatal gut microbiota was as early as the day of birth.

Studies have suggested that the “immune memories” produced by maternal events during pregnancy can have a sustained effect on the development of the offspring’s immune system, thereby assisting in the prevention of the development of allergic diseases (Jenmalm, 2017). Several of these effects depend on maternal antibodies, which may retain microbial-related immune molecules, and are passed to offspring during pregnancy and in breast milk (Vono et al., 2019; Zheng et al., 2020). We found that IgG was spontaneously and significantly increased in the stomach contents (a proxy of breast milk) and serum of neonates. IgG is the only antibody that can cross the placental barrier from mother to offspring. Therefore, it is speculated that the ingestion of exogenous microorganisms during pregnancy, such as the selective probiotic strain TMC3115, may induce the mother to produce specific antibodies and B cells with immune memory, and other microbe-related immune molecules. These can then enter the offspring *via* the placenta and breast milk to induce the offspring to form an immune tolerance to symbiotic bacteria and exogenous microorganisms, and to promote the development of the immune system and gut microbiota.

Weaning (PND 21) was an important turning point for the gut microbiota and host immune and intestinal development. Al Nabhani et al. found that because of continuous microbial and food antigen stimulation during weaning, changes in the gut microbiota and metabolic products such as SCFA can cause a strong immune response; a “weaning reaction” (Al Nabhani et al., 2019). The weaning reaction can be considered as an amplified response of the immune interaction between the microbiota and the host, that is necessary for the formation of tolerance to immune-related diseases in adulthood. The lactation period is also considered to be a key period in the regulation of the gut microbiota (Ronan et al., 2021). In this study, the abundance of *Lactobacillus* was significantly increased both in maternal and offspring gut microbiota at weaning. The crypt depth, colonic epithelial cell proliferation, and expression of tight junction proteins of offspring were significantly increased at weaning, while the levels of IgE were significantly decreased after gestational TMC3115 treatment. The intestine, as the habitat of the gut microbiota and the largest immune tissue in the human body, plays a vital role in microbiota-host immunity interactions (Cheng et al., 2017). IgE is one of the important foundations and biomarkers for the diagnosis of allergic diseases (Owen, 2007), and an increase in IgE can reflect the body’s susceptibility to IgE-mediated allergic diseases, the main category of allergic diseases, to a certain extent (Sohn et al., 2005). Therefore, these results indicated that TMC3115 exposure during pregnancy could promote the development of intestinal tissue and enhance the intestinal barrier function of offspring. These changes may regulate the susceptibility of offspring to IgE-mediated allergic diseases. This effect does not appear in the neonatal period when the immune system is not fully developed, suggesting that it may be related to the changes in intestinal microbes during weaning and the corresponding weaning reaction.

After weaning, the gut microbiota and immune system of offspring continuously changed during puberty (PND 49), although the effects on intestinal development were not persistent. Maternal gestational TMC3115 treatment significantly enhanced the mucosal immunity and ability to regulate inflammation, as evidenced by significantly increased levels of fecal sIgA and serum IL-10 and IL-17A, with dynamic changes in serum immunoglobulin. TMC3115 treatment significantly increased the abundance of *Bacteroidota* and *Muribaculaceae*, whereas those of *Firmicutes* and *Lachnospiraceae* NK4A136 group, and levels of propionic acid, all decreased. Most *Bacteroidota* and *Firmicutes* bacteria, including *Muribaculaceae* and *Lachnospiraceae* NK4A136, are SCFA producers (Koh et al., 2016). A recent study indicated that *Muribaculaceae* were a key taxon responsive to 2-O-β-d-glucopyranosyl-l-ascorbic acid, which can exert an immunomodulatory effect (Huang et al., 2020). The production of acetic acid and alterations found in immune systems may be induced by modifications of the gut microbiota. These results suggested that maternal events inside and outside the uterus, especially those affecting the gut microbiota and its metabolites, may have a remodeling effect on the intestines, microbiota, and immune system of the developing fetus, and continue until birth, with long-term effects.

To further determine whether maternal TMC3115 treatment could affect the susceptibility of offspring to IgE-mediated allergic diseases, and to investigate the underlying mechanisms, an OVA-stimulated model was used, involving offspring from PND 21–PND 49. In this study, the differences in levels of serum IgE and OVA-specific IgE/IgG1 between groups were not significant, indicating that maternal TMC3115 treatment might not affect the susceptibility of offspring to IgE-mediated allergic diseases. However, maternal TMC3115 treatment significantly enhanced the primary immune response to antigens, as shown by the significantly increased level of serum IgM. Similarly, RCT studies have shown that probiotics as adjuvants have good prospects for application in the oral immunotherapy of food allergies, such as those for peanut, egg, and milk protein, with negative results in the levels of total serum IgE (Kim et al., 2010; Tang et al., 2015). The purpose of these applications is to quickly induce immune tolerance to allergens, and to avoid the possible side effects of simple oral food antigen therapy (Loh and Tang, 2018). Therefore, it is speculated that the administration of oral probiotics, such as TMC3115, during pregnancy may induce an immune tolerance to food allergens in the offspring, resulting in insensitivity to the IgE response.

In the OVA-stimulated offspring, which were born from maternal TMC3115-treated dams, intestinal epithelial tissue development, mucin secretion, and intestinal barrier function were significantly enhanced, and the levels of SCFA and abundance of the SCFA-producing bacteria *Lactobacillus* and *Lachnospiraceae* NK4A136 group, were also increased, a finding which is consistent with our previous study, in which neonatal TMC3115 treatment from birth to weaning significantly decreased the susceptibility to IgE-mediated allergic diseases by regulating the gut microbiota and SCFA production (Cheng et al., 2019). SCFA can be used as an energy source for intestinal epithelial cells, to promote the development of intestinal tissues and enhance the intestinal barrier function. SCFA can also interact with G protein-coupled receptors on the surface of immune cells receptors and bind or inhibit the expression of histone deacetylases, to exert their immunomodulatory effects (Koh et al., 2016). A high dietary fiber maternal diet or acetic acid supplementation during pregnancy has been found to reduce the levels of serum cytokines and IgE and lung inflammatory cell infiltration in allergic airway reactions in adult offspring (Thorburn et al., 2015). Kimura et al. further found that maternally derived SCFA produced by a high dietary fiber diet during pregnancy can regulate the development of the intestinal tract and islets of the offspring during the embryonic period, through the maternal gut microbe-SCFA-embryo GPR41/GPR43 axis, to protect the offspring from metabolic diseases such as obesity (Kimura et al., 2020). Therefore, supplementation of selective probiotic strains such as TMC3115 during pregnancy may improve the level of SCFA in offspring in a manner similar to that of high dietary fiber diets, which may enhance the intestinal barrier function and the immune response ability of allergens.

During the long-term co-evolution with the host, bifidobacteria has developed many unique physiological characteristics that are compatible with the host, such as decomposition of HMOs and tolerance to lysozyme in breast milk (Wong et al., 2020). Ecological studies have shown that vertical transmission from mother to offspring is a common ecological feature of bifidobacteria, reflecting the clear evolutionary relationship between *Bifidobacterium* species present in both the mother and offspring (Moeller et al., 2016). A number of population studies have also found that bifidobacteria can be passed from the mother’s vagina, gastrointestinal tract, breast milk, placenta, and amniotic fluid to infants, indicating that the mother is an important source of bifidobacteria found in the infant’s intestines, and that the vertical transmission of bifidobacteria from mother to infant is essential for the normal construction of infant gut microbiota (Wong et al., 2020; Wang et al., 2020). Furthermore, studies have shown that in addition to bifidobacteria, other members of the gut microbiota, such as the certain *Streptococcus*, *Lactobacillus*, and *Lactococcus* specices, may also be transferred from the mother to the infant’s intestines directly or to the breast milk, and then colonized through vertical transmission, thereby affecting the infant’s gut microbiota and immune system development (Perez et al., 2007). In light of this, we inferred that supplementation with TMC3115 during pregnancy can promote the vertical transmission of bifidobacteria and other gut microbiota from mother to infant, thereby promoting the immune development and reducing the risk of allergic diseases in offspring, although the specific involved molecule or cells need to be further clarified.

In conclusion, the maternal gut microbiota, immune function, and intestinal tissue development were fundamentally stable after TMC3115 treatment, whereas the vaginal microbiota was dramatically altered, with a significant increase in alpha diversity and abundance of *Bifidobacterium*. The stomach contents from newborn offspring and the gut microbiota of neonates and weanlings were most changed by maternal TMC3115 treatment, suggesting that these two time points were the important “window of opportunity” for neonatal gut microbiota construction. Synchronous changes in the intestinal, vaginal, and stomach contents (a proxy for breast milk) microbiota were found both in mothers and offspring, involving *Proteobacteria* and *Bifidobacterium*. This observation suggests that the microbiota of maternal gut or breast milk may have a profound modulatory effect on the development of the immune system, intestinal tissue, and gut microbiota composition of offspring. Maternal TMC3115 treatment may induce an immune tolerance to OVA stimulation in offspring, and enhance intestinal tissue development, barrier function, and primary immune response to food allergens. These changes may be related to the significant increases in the relative abundances of *Lactobacillus* and *Lachnospiraceae* NK4A136 group, and in fecal SCFA production. Further research should focus on the role of signaling molecules and potential mechanisms of gut microbiota and their metabolites such as SCFA, that protect the body from immune diseases.

This study has three limitations: ① although the stomach contents of offspring was considered to be a proxy for breast milk, microbiota found in the pups’ stomach contents in our present study might be not fully representative of the microbes in the mother’s breast milk, because the microbiota detected in the pups’ stomach contents might contain the microbes from the pups’ stomach, and the skin microbiota around the nipples or even fecal microbiota. ② Several studies have used feces as the sample origin when detecting gut microbiota, and the fecal microbiota has been considered as the best representative of the gut microbiota. However, in this study, we failed to collect the feces of pups at PND 0, 7, and 14. Hence, we used the intestinal contents as an proxy for feces, although the intestinal contents might contain the microbes resident in the intestinal mucosa, not just the microbes found in feces, making it different from the fecal microbiota of pups at PND 21 and 49. More appropriate methods for sampling the breast milk and feces of young pups are needed to draw a more powerful conclusion. ③ The IgE-mediated allergic disease animal model used in this present study was not a strict allergic diseases animal model, because no allergic symptoms, such as asthma or atopic dermatitis, were observed. The present model was used to evaluate the susceptibility of animals to IgE-mediated allergic diseases, because the main indicators were serum IgE, OVA-specific IgE, and other immune-related indexes, suggesting that considerable further research is needed for pre-clinical studies to be translated to clinical application. Further studies focusing on the preventive/alleviating effects of maternal TMC3115 usage on the offspring’s allergic diseases are needed.

## Data availability statement

The data presented in the study are deposited in the NCBI repository, accession number: PRJNA774623. (https://dataview.ncbi.nlm.nih.gov/object/PRJNA774623?archive=sra).

## Ethics statement

The animal study was reviewed and approved by the animal experiment facility and animals used for the present study were officially approved by the Experimental Animal Management Committee of the Sichuan Government. Experimental protocols were approved by the West China School of Public Health Medical Ethics Committee of Sichuan University (Approval number: SYXK2018-011).

## Author contributions

RC, XS and FH designed this study; RC, YZ and YY conducted the experiment; JL and YW analyzed the data; LR provided the probiotic powder; RC wrote the original manuscript; RC, XS and FH revised the manuscript. All authors contributed to the article and approved the submitted version.

## Funding

This work was supported by the National Natural Science Foundation of China (Grant number: 81973042) and National Science Foundation for Young Scientists of China (Grant number: 82003453) and China Postdoctoral Science Foundation (Grant number:2022M712228).

## Acknowledgments

The authors would like to thank to Chengdu Basebiotech Co., Ltd. for providing assistance on bioinformatics analysis and Enago (http://www.enago.jp) for the English language review. We also appreciate the support of Public health and Preventive Medicine Provincial Experiment Teaching Center at Sichuan University and Food Safety Monitoring and Risk Assessment Key Laboratory of Sichuan Province.

## Conflict of interest

LR is an employee of Hebei Inatural Bio-tech.

The remaining authors declare that the research was conducted in the absence of any commercial or financial relationships that could be constructed as a potential conflict of interest.

The reviewer RH declared a shared affiliation with the authors to the handling editor at the time of review.

## Publisher’s note

All claims expressed in this article are solely those of the authors and do not necessarily represent those of their affiliated organizations, or those of the publisher, the editors and the reviewers. Any product that may be evaluated in this article, or claim that may be made by its manufacturer, is not guaranteed or endorsed by the publisher.
