# Supplementary material for: Maternal gestational Bifidobacterium bifidum TMC3115 treatment shapes construction of offspring gut microbiota and development of immune system and induces immune tolerance to food allergen
Source: Front Cell Infect Microbiol. 2022 Nov 14;12:1045109. doi: 10.3389/fcimb.2022.1045109 (PMC9701730; doi:10.3389/fcimb.2022.1045109)
Supplement: Supplementary file 1 [file DataSheet_1.pdf]

Supplementary material

Maternal gestational *Bifidobacterium bifidum* TMC3115 treatment shapes construction of offspring gut microbiota and development of immune system and induces immune tolerance to food allergen

Ruyue Cheng[1], Yujie Zhang[1], Yang Yang[1], Lei Ren[2], Jinxing Li[1], Yimei Wang[1], Xi Shen[1*], Fang He[1*].

[1] Department of Nutrition and Food Hygiene, West China School of Public Health and West China Fourth Hospital, Sichuan University, Chengdu 610041, Sichuan, P.R. China.

[2] Hebei Inatural Bio-tech Co., Ltd, Shijiazhuang, 050000, Hebei, P.R. China.

Running title: Gestational probiotics treatment shapes offspring gut microbiota and immune function.

*: Correspondence

Fang He

Department of Nutrition and Food Hygiene, West China School of Public Health and West China Fourth Hospital, No. 16, 3rd Section, South Renmin Road, Wuhou District, Chengdu 610041, Sichuan, P.R. China.

Phone: +86-186-0288-0124

E-mail: hf18602880124@163.com

**: Co-Correspondence

Xi Shen

Department of Nutrition and Food Hygiene, West China School of Public Health and

West China Fourth Hospital, Sichuan University, No. 16, 3rd Section, South Renmin Road, Wuhou District, Chengdu 610041, Sichuan, P.R. China.

Phone: +86-152-0821-3847

Email: hxgwshenxi@sina.com

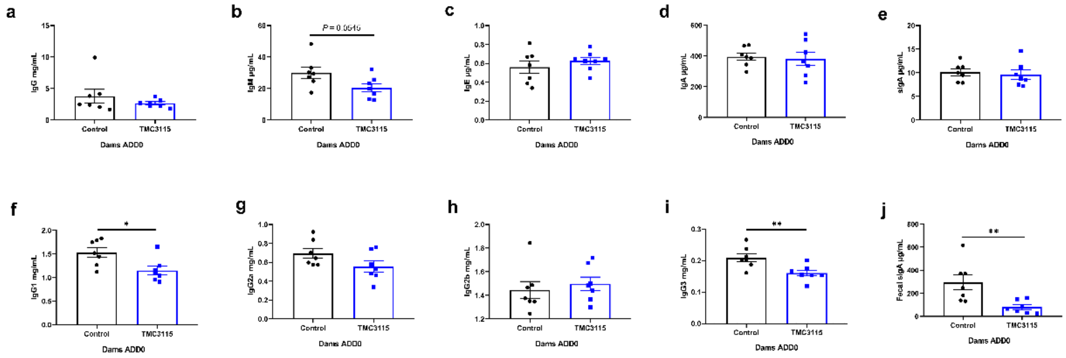

Figure S1 Serum immunoglobulins of dams at ADD0. (a-i) Serum IgG and subclass, IgM, IgE, IgA, sIgA levels of dams at ADD0. (j) Fecal sIgA levels of dams at ADD0. *: $P < 0.05$, **: $P < 0.01$, compared with Control. ADD: after delivery day. $n = 7$/group.

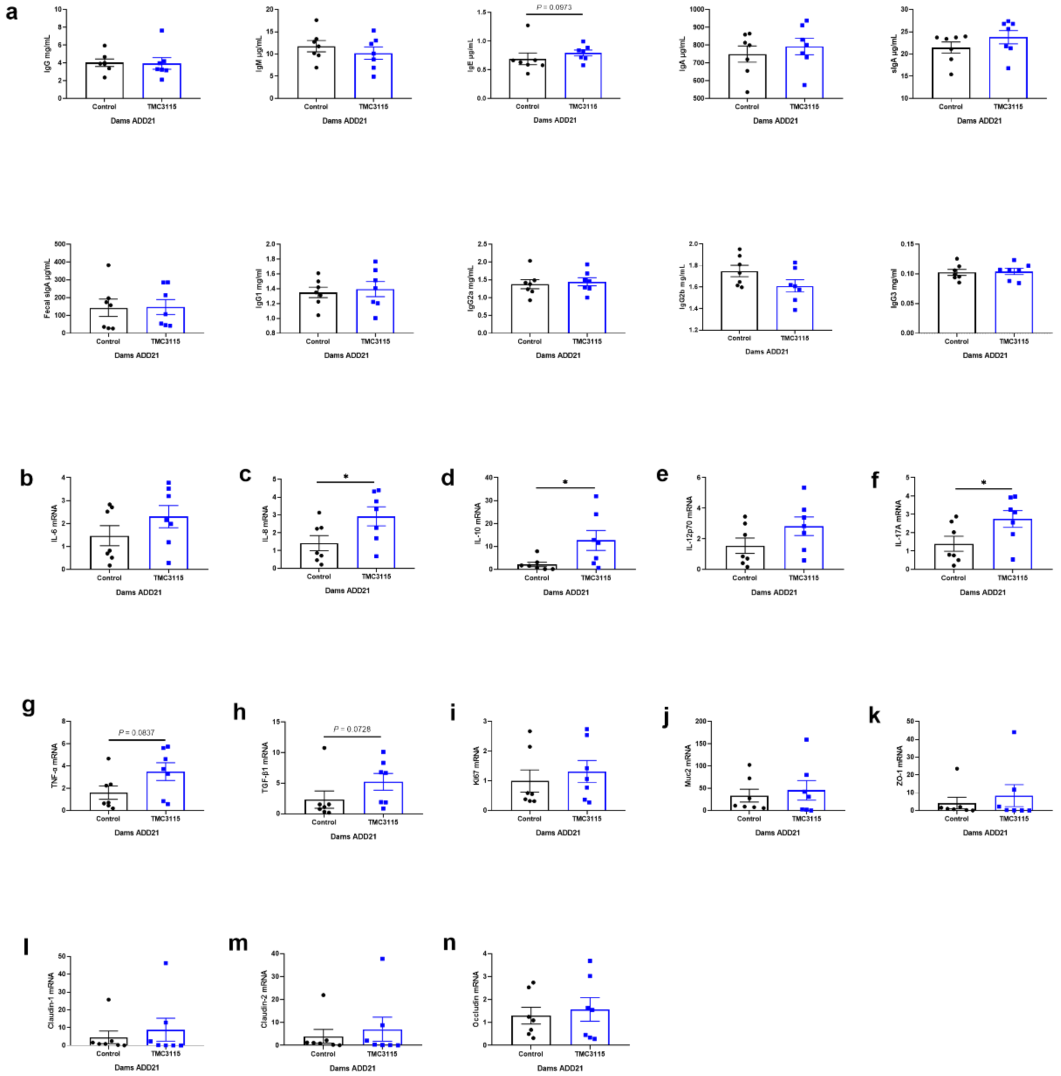

Figure S2 Serum immunoglobulins, splenic cytokines mRNA expression and intestinal tissue development of dams at ADD21. (a) Serum IgG and subclass, IgM, IgE, IgA, sIgA and fecal sIgA levels of dams at ADD21. (b-h) Splenic cytokines mRNA expression of dams at ADD21. (i-n) Colonic Ki67, Muc2 and tight junction proteins mRNA expression of dams at ADD21. *: $P < 0.05$, compared with Control. ADD: after delivery day. $n = 7$/group.

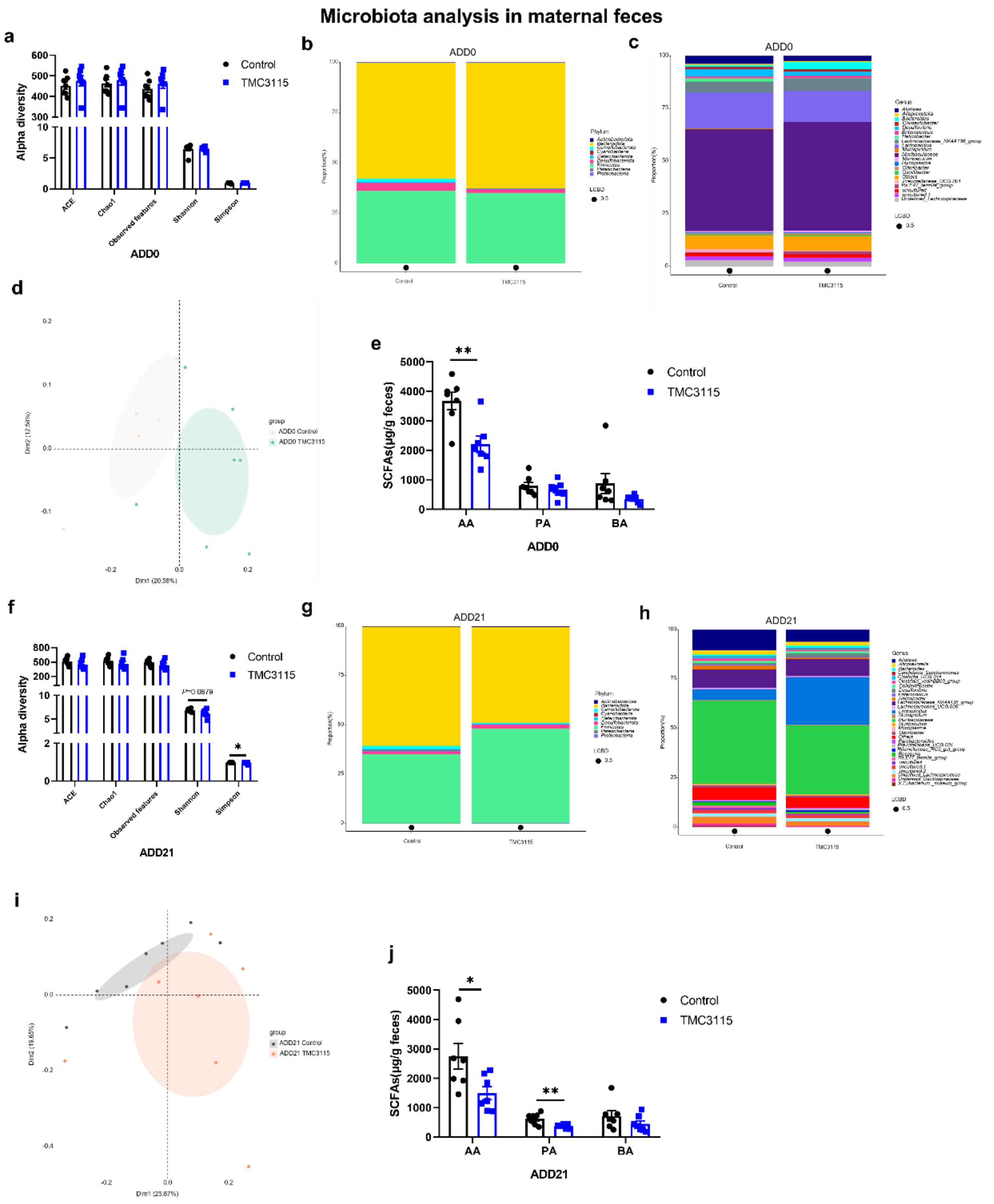

Figure S3 Diversity and composition of maternal fecal microbiota at ADD0 and ADD21. (a, f) Alpha diversity of dams at ADD0 and ADD21. (b-c, g-h) The bar plot of maternal fecal microbial community at phylum and genus level of dams at ADD0 and ADD21. (d, i) PCoA analysis based on bray-curtis distance of dams at ADD0 and ADD21. (e, j) Fecal SCFAs production of dams at ADD0 and ADD21. *: $P < 0.05$, **: $P < 0.01$, compared with Control. ADD: after delivery day. AA: acetic acid; PA: propionic acid; BA: butyric acid. $n = 7$/group.

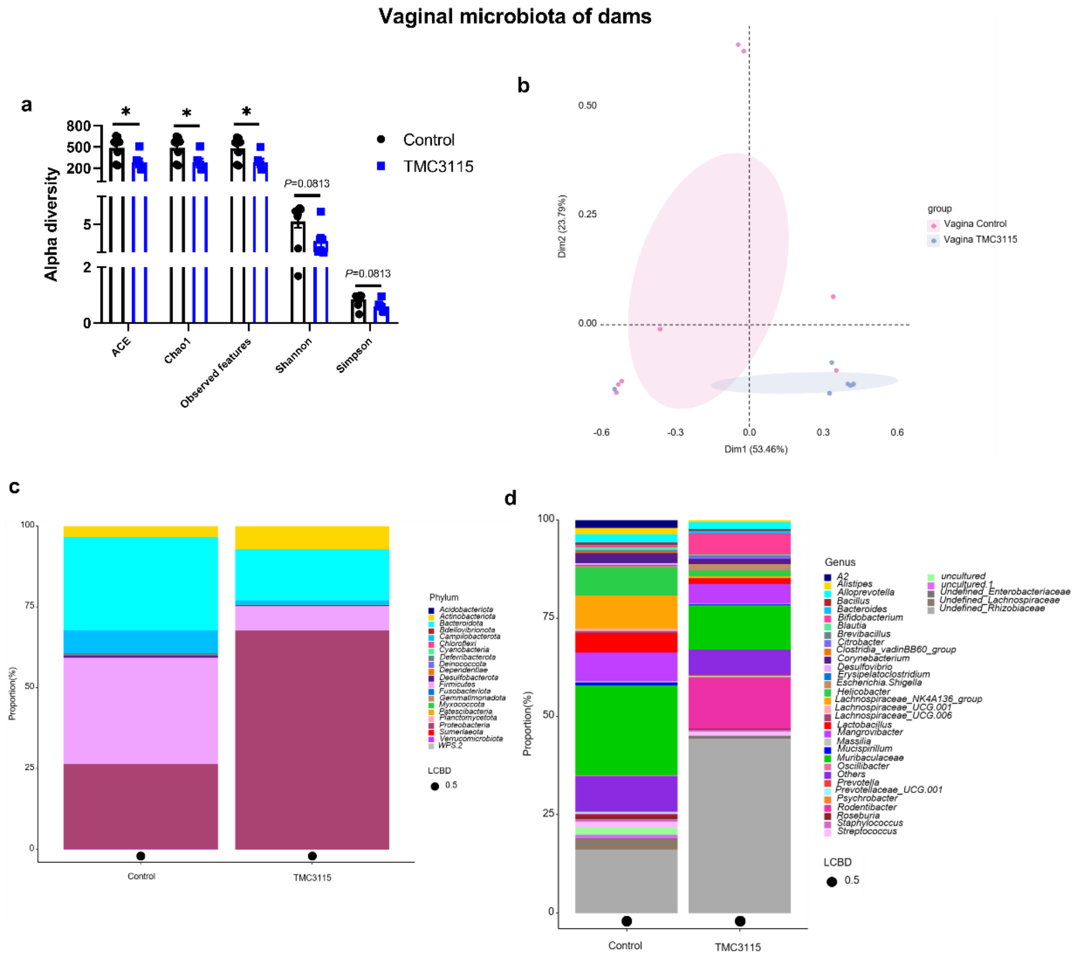

Figure S4 Vaginal microbiota of dams at ADD0. (a) Alpha diversity of vaginal microbiota of dams at ADD0. (b) PCoA analysis based on bray-curtis distance of vaginal microbiota of dams at ADD0. (c-d) The bar plot of vaginal microbial community at phylum and genus level of dams at ADD0. *: $P < 0.05$, compared with Control. ADD: after delivery day. Control group, $n = 8$; TMC3115 group, $n = 6$.

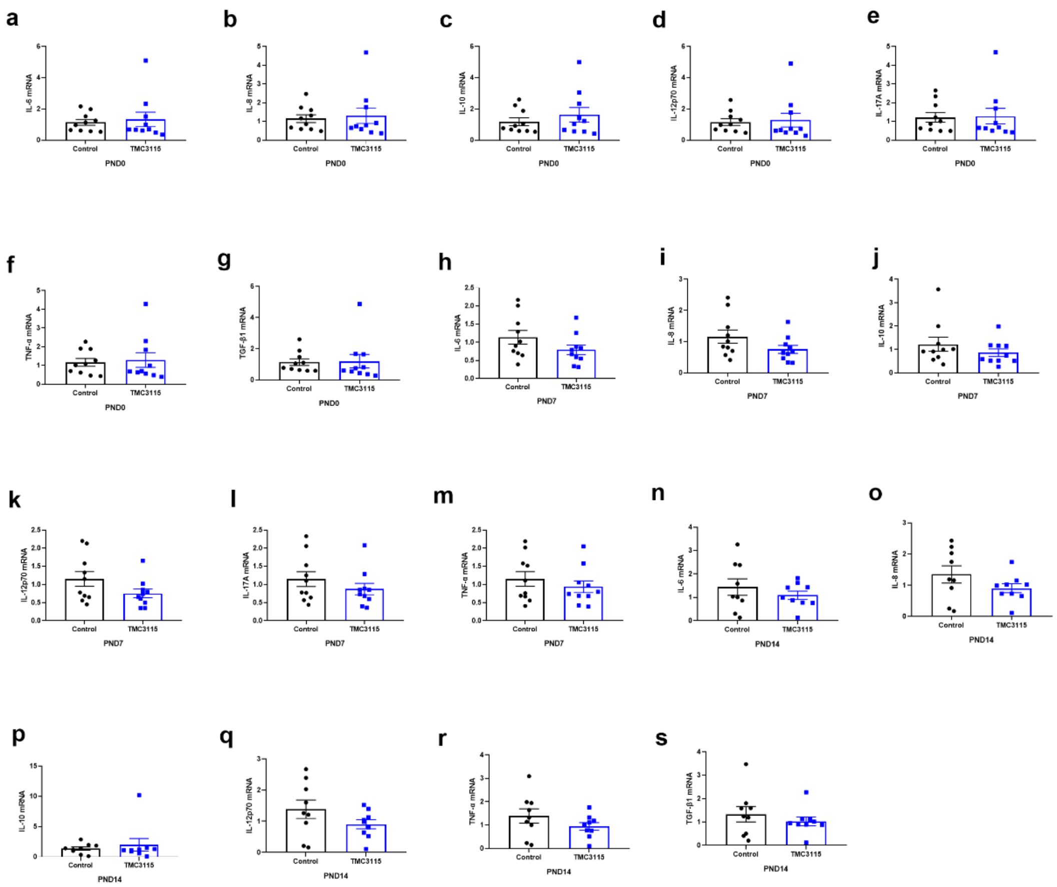

Figure S5 Splenic cytokines mRNA expression of offspring from PND0-14. (a-s) Similar changes in splenic cytokines mRNA expression of offspring at PND0, PND7 and PND14 between two groups. PND: postnatal day. n = 9-10/group.

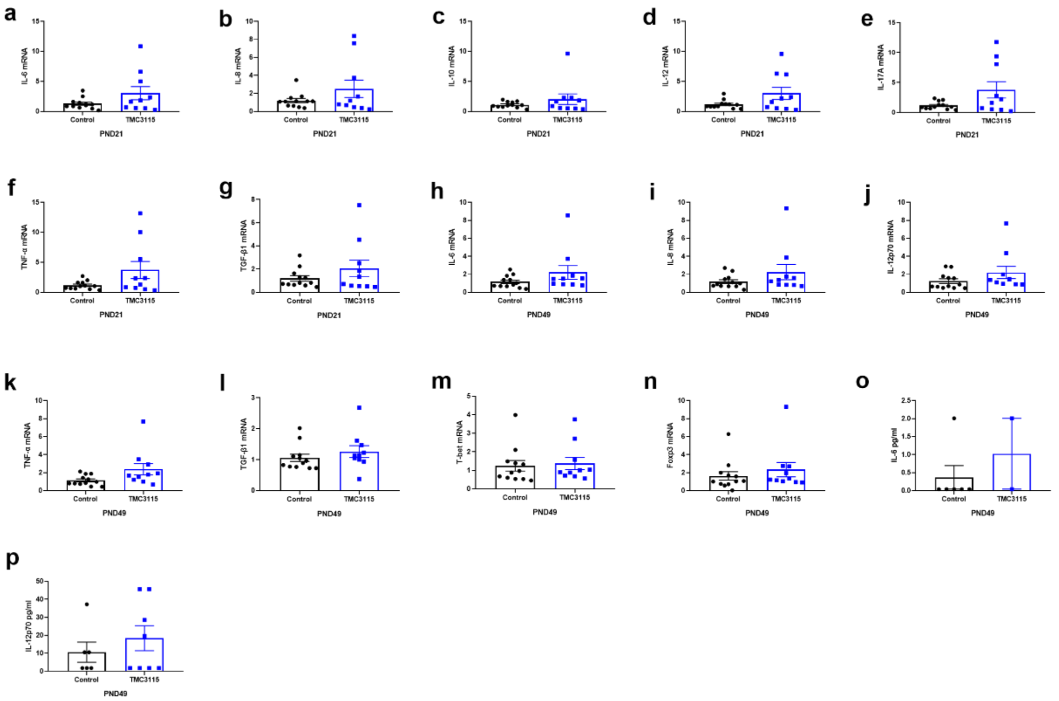

Figure S6 Splenic cytokines mRNA expression of offspring from PND21-49. (a-p) Similar changes in splenic cytokines mRNA expression of offspring at PND21 and PND49 between two groups. PND: postnatal day. *n* = 6-12/group.

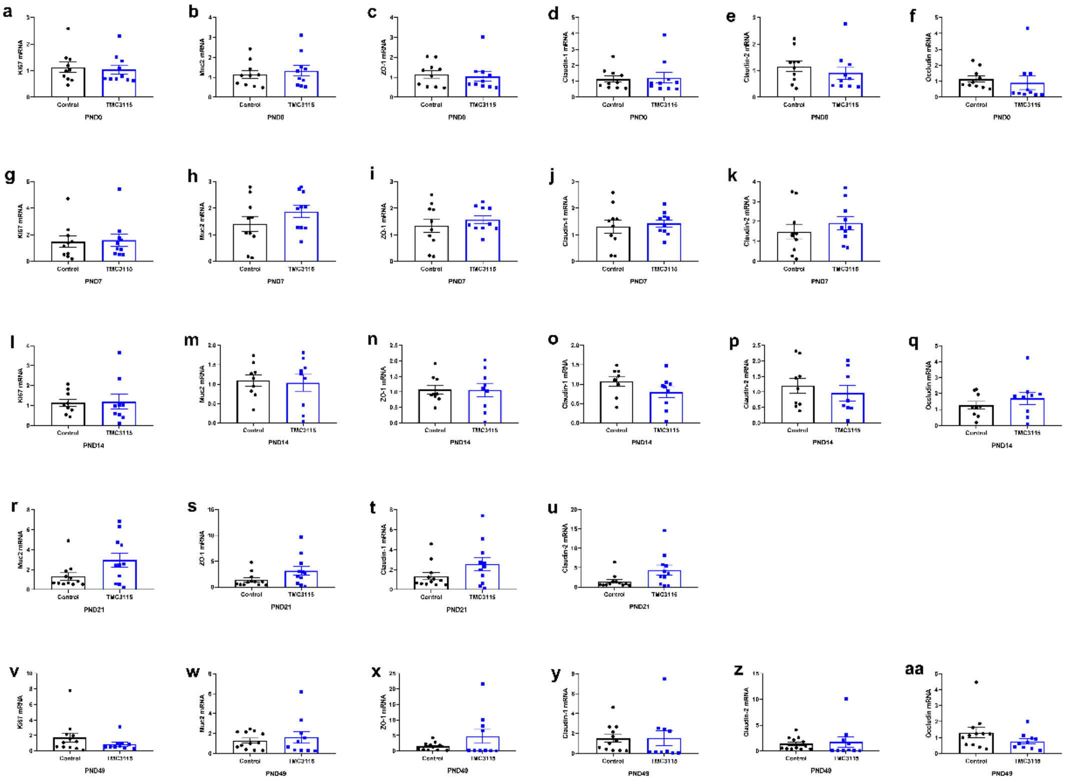

Figure S7 Colonic intestinal tissue development indicators mRNA expression of offspring from

PND0-49. (a-aa) Similar changes in colonic intestinal tissue development indicators mRNA expression of offspring at PND0-49 between two groups. PND: postnatal day. n = 9-12/group.

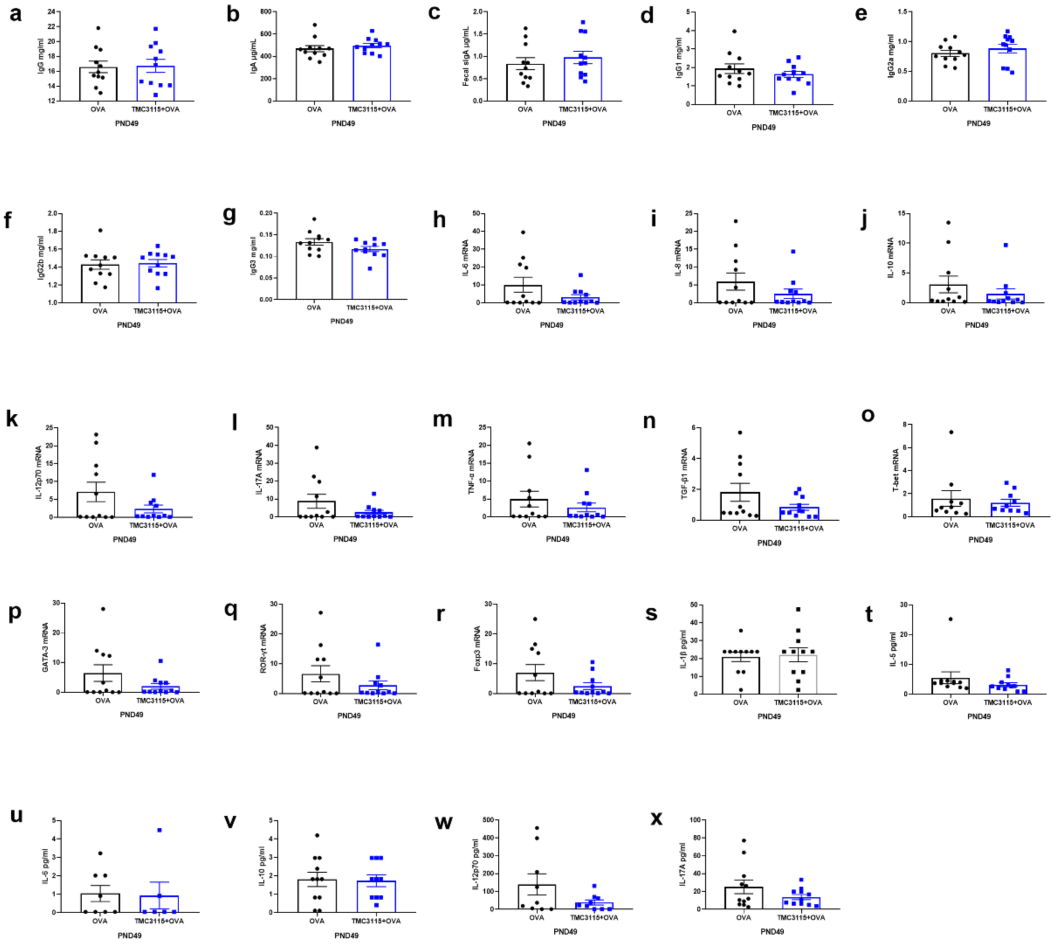

Figure S8 Serum immunoglobulins and cytokines and splenic cytokines mRNA expression of offspring after OVA stimulation at PND49. (a-g) Similar changes in serum IgG and subclass, IgA and fecal sIgA levels of offspring after OVA stimulation at PND49 between two groups. (h-r) Similar changes in splenic cytokines and transcriptional factors mRNA expression between groups. (s-x) Similar changes in serum cytokines levels between groups. PND: postnatal day. n = 11/group.

Table S1 Sequences of primers

Primer	Sequence (5'~3')
β-actin-F	GTGGGCCGCTCTAGGCACCAA
β-actin-R	CTCTTTGATGTCACGCACGATTTC
IL-6-F	GTCACAGAAGGAGTGGCTA
IL-6-R	AGAGAACAACATAAGTCAGATACC
IL-10-F	GACCAGCTGGACAACATACT
IL-10-R	GAGGGTCTTCAGCTTCTCAC
IL-12p70-F	CTCTGTCTGCAGAGAAGGTC
IL-12p70-R	GCTGGTGCTGTAGTTCTCAT
IL-17A-F	TGATGCTGTTGCTGCTGCTGAG
IL-17A-R	CACATTCTGGAGGAAGTCCTTGGC
T-bet -F	ATCACTAAGCAAGGACGGCGAATG
T-bet -R	TCCACCAAGACCACATCCACAAAC
GATA-3-F	CCTCTCCTTCGGACCTCACCAC
GATA-R	CGGAGGAACTCTTCGCACACTTG
ROR-γt-F	TGTCCCGAGATGCTGTCAAGTTTG
ROR-γt-R	TCCTGTTGCTGCTGCTGTTGC
FOXP3-F	TGCGAGTGGAGAGCGAGAAGG
FOXP3-R	AGGTCAAGGGCAGGGATTGGAG
Claudin-1-F	GCTGGGTTTCATCCTGGCTTCTC
Claudin-1-R	CCTGAGCGGTCACGATGTTGTC
Claudin-2-F	CACCGTGTTCTGCCAGGATTCTC
Claudin-2-R	TCAGGAACCAGCGGCGAGTAG
Occludin-F	GCGAGGAGCTGGAGGAGGAC
Occludin-R	CGTCGTCTAGTTCTGCCTGTAAGC
KI67-F	GCCTGCCCGACCCTACAAAATG
KI67-R	CTCATCTGCTGCTGCTTCTCCTTC
MUC2-F	TGCTGACGAGTGGTTGGTGAATG
MUC2-R	TGATGAGGTGGCAGACAGGAGAC
ZO-1-F	GCGAACAGAAGGAGCGAGAAGAG
ZO-1-R	GCTTTGCGGGCTGACTGGAG

Note: F: forward primer, R: reverse primer.

Table S2 Mean relative abundance of maternal fecal and vaginal microbiota at phylum and genus level（%）

Stage	Phylum/Genus	Control	TMC3115
Vagina	*Firmicutes*	32.63	7.42*
	Proteobacteria	27.09	69.72*
	Desulfobacterota	0.80	0.15*
Vagina	*Oscillibacter*	0.44	0.03**
	A2	2.09	0.03**
	Lachnospiraceae.UCG.006	0.46	0.02*
	Clostridia.vadinBB60.group	0.35	0.03*
	Desulfovibrio	0.47	0.04*
	Lachnospiraceae.NK4A136.group	8.58	0.45*
	Escherichia.Shigella	0.55	1.76*
	Bifidobacterium	0.43	6.14*
	Citrobacter	0.12	0.78*
	Undefined.Lachnospiraceae	2.65	0.20*
	Lactobacillus	4.94	1.25*
	Psychrobacter	0.00	0.21*
ADD0	*Proteobacteria*	5.86	2.52*
	Deferribacteres	0.65	0.13*
ADD0	*Helicobacter*	4.43	0.75**
	Mucispirillum	1.72	0.38*
	Prevotella	3.95	0.67*
	Rikenella	0.26	0.48*
ADD21	*Alistipes*	29.11	14.43*
	Lactobacillus	17.64	45.42**
	Roseburia	1.97	0.45**
	Staphylococcus	0.16	0.57*

Note: ADD: after delivery day; *: compared with Control, $P < 0.05$; **: compared with Control, $P < 0.01$; for fecal samples, $n = 7$/group; for vaginal douche, Control group: $n = 8$, TMC3115 group, $n = 6$. Mann-Whitney test was used for pairwise comparison.

Table S3 Body weight of the tested mice during different life stages (g)

Stage		Control	TMC3115
Dams	Before experiment	32.64±2.02	34.62±2.20
	ADD0	29.99±1.71	29.67±1.88
	ADD21	29.69±1.77	29.30±1.81
Pups	PND0	1.40±0.17	1.37±0.22
	PND7	5.31±0.53	5.24±1.10
	PND14	11.72±1.27	11.56±1.91
	PND21	12.49±0.68	11.73±2.02
	PND49	22.13±1.87	26.03±2.82***
	PND49 (OVA-stimulated)	24.11±2.87	25.08±1.84

Note: data was shown as mean±SD. ADD: after delivery day; PND: postnatal day; ***: compared with Control, $P < 0.001$; Before experiment, Control group: n = 7, TMC3115 group, n = 9; ADD0 and ADD21, n = 7/group; PND0, Control group: n = 78, TMC3115 group, n = 71. PND7, Control group: n = 41, TMC3115 group, n = 37; PND14, Control group: n = 22, TMC3115 group, n =19; PND21, n = 12/group; PND49, Control group: n = 12, TMC3115 group, n =10; PND49 (OVA-stimulated), n = 11/group; Student T test was used for pairwise comparison.
